# Hypopituitarism Revealing Probable Neurosarcoidosis: A Case Report and Diagnostic Challenges

**DOI:** 10.3390/reports9020113

**Published:** 2026-04-07

**Authors:** Michał Szklarz, Mikołaj Madeksza, Katarzyna Wołos-Kłosowicz, Julia Modzelewska, Jan Górny, Wojciech Matuszewski

**Affiliations:** Clinic of Endocrinology, Diabetology and Internal Medicine, School of Medicine, Collegium Medicum, University of Warmia and Mazury in Olsztyn, 10-957 Olsztyn, Polandjulia.modzelewska12@gmail.com (J.M.);

**Keywords:** neurosarcoidosis, sarcoidosis, hypopituitarism, hypogonadism

## Abstract

**Background and Clinical Significance:** Neurosarcoidosis (NS) is a rare manifestation of systemic sarcoidosis involving the central nervous system, with highly variable neurological and endocrine presentations. Among these, anterior pituitary dysfunction is particularly uncommon and diagnostically challenging. **Case Presentation:** We report the case of a 37-year-old woman with a 4-year history of secondary amenorrhoea and an initially suspected pituitary microadenoma, who was ultimately diagnosed with probable NS presenting with multiaxial anterior pituitary insufficiency. Early magnetic resonance imaging (MRI) revealed a small pituitary lesion and isolated pituitary stalk thickening, without other central nervous system abnormalities. Subsequent imaging demonstrated contrast-enhancing lesions involving the meninges and cranial nerves, along with progression of pituitary stalk involvement and loss of the posterior pituitary bright spot. Further evaluation confirmed systemic sarcoidosis. High-dose corticosteroid therapy led to partial clinical and radiological improvement; however, relapse necessitated methotrexate, and persistent pituitary hormone deficiencies required long-term hormonal replacement. **Conclusions:** This case highlights the diagnostic complexity of NS presenting with isolated endocrine dysfunction and subtle imaging findings. It underscores the need to consider systemic sarcoidosis in patients with unexplained hypopituitarism.

## 1. Introduction and Clinical Significance

Sarcoidosis is a systemic disease characterised by the formation of non-caseating granulomas in affected organs. Its aetiology remains unknown; however, it is thought to involve an exaggerated immune response to unidentified antigens, triggered by genetic and environmental factors [1,2,3]. The global incidence of sarcoidosis ranges from 1 to 35 per 100,000, and in Poland, it is estimated at 10 per 100,000 persons per year [3,4].

Neurosarcoidosis (NS) is a less common manifestation of sarcoidosis, resulting from granulomatous involvement of the central and/or peripheral nervous systems. Its clinical and imaging manifestations are highly variable. In a meta-analysis by Sambon et al., the most frequent findings were meningeal involvement (48%), parenchymal lesions (47%), myelopathy/spinal cord involvement (34%), and cranial nerve palsy (33%) [5]. Most patients have multifocal lesions. The clinical diagnostic criteria for NS are shown in Table 1 [6].

Hypothalamic-pituitary involvement occurs in approximately 11% of patients with NS and may affect both anterior and posterior pituitary function [5,7]. Hypogonadotropic hypogonadism and diabetes insipidus account for more than half of endocrine manifestations, followed by central hypothyroidism and prolactin abnormalities, while ACTH and GH deficiencies are the least common [8,9]. The prevalence of individual disorders is summarised in Table 2.

## 2. Case Presentation

We describe a 37-year-old woman in whom probable NS was identified as the underlying cause of multiaxial anterior pituitary dysfunction. She was admitted to the Endocrinology Department in October 2023. Secondary amenorrhoea had been diagnosed in 2019. Pituitary MRI performed in 2020 showed a 2 mm hypointense lesion at the border of the lobes and focal thickening of the superior pituitary stalk up to 5 mm, with otherwise normal gland dimensions. A microadenoma was initially suspected, and bromocriptine therapy was started but discontinued due to lack of clinical effect. Key laboratory findings are summarised in Table 3. Other laboratory parameters, including inflammatory markers, liver function tests, and haematological indices, remained within normal limits throughout the observation and showed no significant changes over time.

**Figure 1:**
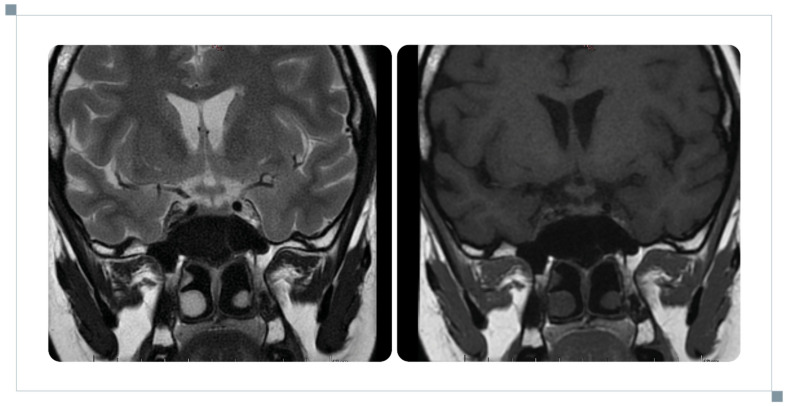
Image of pituitary neurosarcoidosis in our patient during the first hospitalisation.

**Figure 2:**
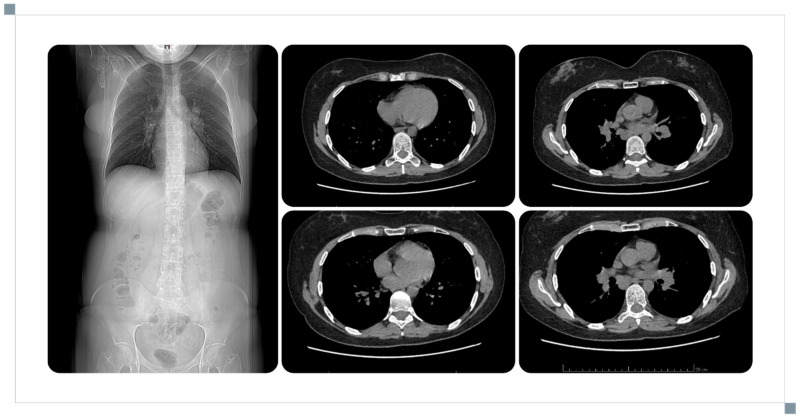
Chest CT scan with lesions typical of sarcoidosis.

On admission, she reported persistent headaches and dizziness for 7 weeks, accompanied by arthralgia and recurrent fever up to 38.8 °C. MRI performed during hospitalisation revealed multiple gadolinium-enhancing lesions involving both optic nerves, as well as the left vagus and trigeminal nerve regions. Additional enhancement was present in the meninges, including the base of the frontal lobe, the right midbrain surface, and the supratentorial region. Brainstem involvement included the pons and medulla oblongata. The posterior pituitary bright spot was absent on non-contrast T1-weighted imaging, and the pituitary stalk was thickened to 5.5 mm over an 8 mm segment (Figure 1). Endocrine evaluation revealed hypogonadotropic hypogonadism and moderate hyperprolactinaemia with a loss of diurnal variation. Assessment of the corticotropic axis showed low DHEA-S and low-normal ACTH, with normal cortisol levels and a preserved response to ACTH stimulation. No polyuria or polydipsia was reported. Overall, the findings were consistent with hypogonadotropic hypogonadism and hyperprolactinaemia. Possible subclinical secondary adrenal insufficiency was considered based on low DHEA-S and ACTH levels, and hydrocortisone was recommended for use in stressful situations (e.g., infection, trauma, or surgery). No other anterior or posterior pituitary dysfunction was evident at that stage. CSF analysis revealed elevated protein and decreased glucose. Extensive evaluation for infectious, autoimmune, and haematological conditions was negative. Chest CT showed bilateral hilar and mediastinal lymphadenopathy with small pulmonary nodules, consistent with stage II pulmonary sarcoidosis (Figure 2). Endobronchial ultrasound-guided fine-needle aspiration of subcarinal lymph nodes demonstrated non-caseating granulomas, confirming the diagnosis. A diagnosis of probable NS was established, and treatment with prednisone 70 mg daily was initiated.

**Figure 3:**
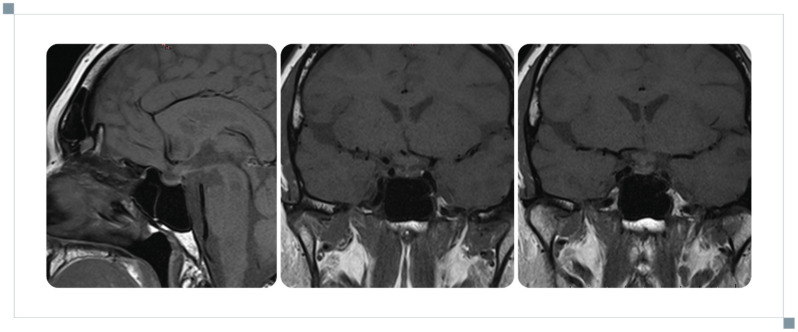
Follow-up MRI of the pituitary gland illustrating partial regression of pathological enhancements and thickening of the infundibulum.

After less than one month, the patient was readmitted to the ward to monitor her therapy. She developed polyuria of up to 6–8 L/day; however, a water deprivation test excluded diabetes insipidus. Follow-up MRI showed partial regression of contrast enhancement and reduction in pituitary stalk thickening (Figure 3). Repeat endocrine testing confirmed persistent hypogonadotropic hypogonadism, and the thyrotropic axis showed central hypothyroidism (low FT3 and FT4 levels with inappropriately low TSH), with a normal TRH response. Hyperprolactinaemia resolved with restoration of the physiological circadian rhythm. At this stage, evolving multiaxial anterior pituitary dysfunction became evident, with involvement of the gonadotropic and thyrotropic axes, while posterior pituitary function remained preserved. Levothyroxine 25 µg/day and hormone replacement therapy were initiated.

**Figure 4:**
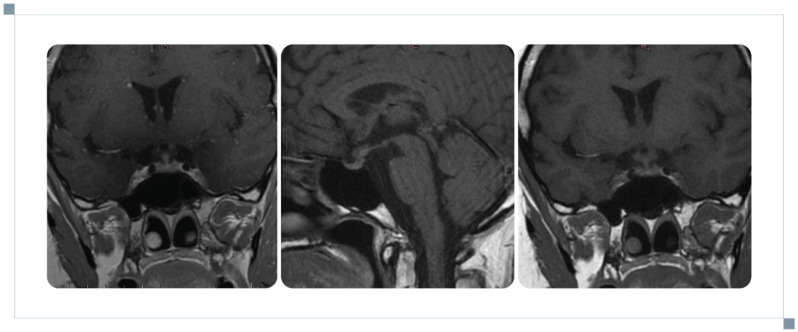
Progression of pathological enhancements within the CNS after a reduction in glucocorticoid dosage.

During glucocorticoid tapering, neurological symptoms recurred, including weakness, dizziness, and impaired concentration. MRI in April 2024 demonstrated progression of CNS lesions (Figure 4). Endocrine reassessment confirmed central hypogonadism and hypothyroidism. A glucagon stimulation test showed inadequate GH response, consistent with somatotropic deficiency. These findings confirmed progression to multiaxial anterior hypopituitarism. Prednisone therapy was adjusted to 7.5 mg/day. Following a pulmonology consultation in May 2024, methotrexate was initiated (10 mg/week), and the prednisone dose was increased to 10 mg/day. Chest CT showed stable pulmonary interstitial changes with partial regression of lymphadenopathy. In June 2024, prednisone and methotrexate doses were increased to 12.5 mg daily and 15 mg weekly, respectively. Recombinant growth hormone therapy was introduced in July 2024. Due to poor glucocorticoid tolerance, methotrexate was further increased to 15 mg/week, and hydrocortisone substitution of 5–10 mg/day was included.

At follow-up 12 months later, the patient’s general condition was stable. She reported no respiratory symptoms or excessive somnolence and achieved intentional weight loss (8 kg/6 months). Menstrual function did not recover. Residual symptoms included hand paraesthesia and papular skin lesions. Laboratory evaluation showed normal IGF-1, persistent gonadotropic and corticotropic insufficiency, and borderline elevated total calcium with normal corrected calcium.

Pulmonary function tests remained within normal limits. Chest CT demonstrated slight progression of pulmonary lesions and new previously unobserved splenic lesions, with regression of lymphadenopathy. CNS imaging showed no lesions in the medulla or spinal cord. Due to progression of pulmonary lesions, methotrexate dosage was increased from 15 to 25 mg/week. At the most recent follow-up, the patient remained on methotrexate 25 mg/week, hydrocortisone 5 mg twice daily, levothyroxine 88 µg/day, recombinant GH 0.4 mg/day s.c., and hormone replacement therapy, with good tolerance and no reported adverse effects. No formal psychological assessment was conducted during the course of the disease, and the patient did not report symptoms such as insomnia, mood disturbance, or loss of appetite. The disease course is summarized in Table 4.

## 3. Discussion

Among the aetiologies of hypopituitarism, NS is one of the rarest. To date, just over 200 cases of hypothalamic-pituitary involvement have been reported [6,9,10,11,12,13,14,15,16,17,18,19,20,21,22,23,24,25,26]. The case we describe illustrates the difficulties often encountered in the diagnosis and treatment of NS.

NS can present with a wide range of clinical and imaging manifestations and is often not initially considered in the differential diagnosis. Establishing a diagnosis, therefore, requires a high degree of clinical suspicion. This challenge is less pronounced in patients with symptomatic extra-neural sarcoidosis, where suspicion of NS arises more readily. Although extra-neural disease is present in the majority of cases, neurological symptoms frequently precede systemic manifestations [7]. This may delay evaluation for systemic sarcoidosis, particularly when CNS findings are non-specific. The clinical course observed in our patient illustrates this challenge. For 4 years, her only manifestations were endocrine disorders and pituitary lesions, which is an uncommon presentation and did not raise suspicion of NS [7,12]. Importantly, the initial MRI in 2020 demonstrated only a small intrasellar lesion and isolated pituitary stalk thickening, without meningeal, cranial nerve, or parenchymal involvement. The more characteristic contrast-enhancing lesions appeared only in 2023. The early clinical picture—secondary amenorrhoea with a focal pituitary lesion and hyperprolactinaemia—led to an initial diagnosis of prolactin microadenoma. However, this represented a diagnostic pitfall: the presence of pituitary stalk thickening and hypogonadotropic hypogonadism disproportionate to the degree of hyperprolactinaemia was atypical for a microprolactinoma and should have prompted consideration of an inflammatory or infiltrative process.

The differential diagnosis of pituitary stalk thickening and hypopituitarism is broad and includes inflammatory, neoplastic, and infectious conditions such as lymphocytic hypophysitis, germinoma, Langerhans cell histiocytosis, tuberculosis, IgG4-related disease, and other granulomatous or infiltrative disorders. In our patient, these possibilities were systematically evaluated. Extensive infectious workup, including CSF cultures, PCR testing for *M. tuberculosis*, and serological testing for neurotropic pathogens, was negative. Autoimmune and inflammatory conditions were considered unlikely given negative ANA, ANCA, IgG4, and antiphospholipid antibody panels, as well as the absence of characteristic systemic features. Neoplastic processes such as germinoma or lymphoma were not supported by imaging findings or CSF analysis. Importantly, the presence of biopsy-proven systemic sarcoidosis, together with a consistent clinical, radiological, and therapeutic course of the CNS disease, supported the diagnosis of probable NS, making the coexistence of two independent pathologies unlikely.

Another notable feature in this case was the dissociation between radiological findings and clinical symptoms. Despite multiaxial anterior pituitary insufficiency and loss of the posterior pituitary bright spot, the patient did not develop diabetes insipidus. Similarly, bilateral optic nerve enhancement was not associated with visual impairment. Such discrepancies have been reported and reflect the heterogeneous and often subclinical involvement of affected structures [5,7,27].

Initial treatment consisted of high-dose glucocorticoids, resulting in rapid clinical and radiological improvement. However, disease relapse during glucocorticoid tapering required the introduction of methotrexate as a steroid-sparing agent, which is a common approach in relapsing or refractory NS [2,28].

This case underscores the importance of considering NS even in the absence of typical neurological symptoms or characteristic radiologic findings. The clinical variability of NS may lead to misdiagnosis, with correct identification often occurring only after disease progression or the onset of symptomatic extra-neural sarcoidosis [29,30]. Early recognition of atypical features and systematic evaluation for systemic sarcoidosis are therefore essential to reduce diagnostic delay and enable timely initiation of immunosuppressive therapy [28].

## 4. Conclusions

NS may present primarily with endocrine dysfunction, and isolated anterior pituitary involvement may delay diagnosis in the absence of overt neurological features.

Thorough evaluation and a high index of clinical suspicion are essential. In patients with unexplained hypopituitarism and pituitary stalk abnormalities, systemic sarcoidosis should be actively investigated, including appropriate imaging and histopathological confirmation where feasible.

Contrast-enhancing lesions involving the meninges, cranial nerves, and pituitary stalk are important diagnostic clues, although MRI findings may initially be subtle and nonspecific.

Corticosteroids remain the first-line treatment, while second-line agents such as methotrexate are often necessary in relapsing disease, alongside appropriate hormonal replacement.

## Figures and Tables

**Table 1:** Diagnostic Criteria for Central Nervous System and Peripheral Nervous System Neurosarcoidosis.

Possible	1. The clinical presentation and diagnostic evaluation suggest neurosarcoidosis, as defined by the clinical manifestations and MRI, CSF, and/or EMG/NCS findings typical of granulomatous inflammation of the nervous system and after rigorous exclusion of other causes. 2. There is no pathologic confirmation of granulomatous disease.
Probable	1. The clinical presentation and diagnostic evaluation suggest neurosarcoidosis, as defined by the clinical manifestations and MRI, CSF, and/or EMG/NCS findings typical of granulomatous inflammation of the nervous system after rigorous exclusion of other causes. 2. There is pathologic confirmation of systemic granulomatous disease consistent with sarcoidosis.
Definite	1. The clinical presentation and diagnostic evaluation suggest neurosarcoidosis, as defined by the clinical manifestations and MRI, CSF, and/or EMG/NCS findings typical of granulomatous inflammation of the nervous system after rigorous exclusion of other causes. 2. The nervous system pathology is consistent with neurosarcoidosis. Type a. Extraneural sarcoidosis is evident. Type b. No extraneural sarcoidosis is evident (isolated CNS sarcoidosis).

Abbreviations: CSF, cerebrospinal fluid; EMG, electromyogram; MRI, magnetic resonance imaging; NCS, nerve conduction study.

**Table 2:** Incidence of respective pituitary disorders in major review papers.

Disorder	Martin-Grace et al. [9], 2015 *N* = 64	Langrand et al. [8], 2012 *N* = 43
Diabetes insipidus	57% (*n* = 37)	60% (*n* = 26)
Hypogonadotropic hypogonadism	46% (*n* = 30)	88% (*n* = 38)
Hypo-/hyperprolactinaemia	27% (*n* = 17)	53% (*n* = 23)
TSH deficiency	24% (*n* = 15)	56% (*n* = 24)
ACTH deficiency	3% (*n* = 2)	37% (*n* = 16)
GH deficiency	-	23% (*n* = 10)

N—number of patients included in the study, n—number of patients meeting a criterion.

**Table 3:** Selected laboratory test results.

Measurement (Unit) [ref.]	January 2020	December 2021	January 2023	13–15 October 2023	17–20 October 2023	November 2023	April 2024
17-OHP (mg/dL)	-	-	-	-	0.04	-	-
Oestradiol (ng/mL)	-	<5.0	-	8.8	-	<5.0	-
FSH (mIU/mL)	-	1.93	-	1.4	-	1.7	-
LH (mIU/mL)	-	-	-	<0.1	-	-	0.6
PRL (µg/L) [4.79–23.31].	-	33.77	-	6 p.m.: 26.14 4 a.m.: 27.61 10 a.m.: 29.22 12 p.m.: 27.62	-	5 p.m.: 9.79 4 a.m.: 16.39 8 a.m.: 21.24 12 p.m.: 11.51	5 p.m.: 26.59 4 a.m.: 38.83 10 a.m.: 36.57 12 p.m.: 31.84
Testosterone (ng/dL) [0.06–0.82].	-	-	-	<0.03	-	-	-
ACTH (pg/mL)	4.8	-	-	-	9 a.m.: 11.9 12 a.m.: 9.4	-	-
DHEA-S (µg/dL) [60.9–337].	-	-	-	13.5	-	9.8	-
cortisol (µg/dL)	14.44	-	-	6 p.m.: 6.8 10 a.m.: 10.94	12 a.m.: 6.02	-	-
TSH (µIU/mL) [0.27–4.2].	-	-	1.9	-	1.39	1.77	1.3; 0.41
FT3	-	-	1.74; ref [1.8–4.6].	-	-	2.9 (pmol/L); ref [3.1–6.8].	4.1 (pmol/L); ref [3.1–6.8].
FT4	-	-	0.782; ref [0.93–1.7].	-	-	11.7 (pmol/L); ref [12–22].	18.8 (pmol/L); ref [12–22].
CSF protein (mg/dL) [14–45].	-	-	-	-	78	-	-
CSF glucose (mg/dL) [40–75].	-	-	-	-	30	-	-
pleocytosis in CSF (cells/µL) [0–4].	-	-	-	-	4	-	-

Abbreviations: 17-OHP, 17-hydroxyprogesterone; ACTH, adrenocorticotropic hormone; CSF, cerebrospinal fluid; DHEA-S, dehydroepiandrosterone sulfate; FSH, follicle-stimulating hormone; FT3, free triiodothyronine; FT4, free thyroxine; LH, luteinizing hormone; PRL, prolactin; TSH, thyroid-stimulating hormone.

**Table 4:** Chronological summary of clinical course, imaging findings, endocrine results, treatment, and outcomes.

Time	Clinical Features	MRI Findings	Endocrine Findings	Treatment	Outcome
2020	Secondary amenorrhoea	2 mm intrasellar lesion; pituitary stalk thickening (5 mm)	Low gonadotropins; low oestradiol	Bromocriptine	No clinical improvement
October 2023	Headache, dizziness, fever, arthralgia	Meningeal, cranial nerve, and brainstem contrast-enhancing lesions; pituitary stalk thickening; absent posterior pituitary bright spot	Low LH/FSH; low oestradiol; elevated prolactin; low DHEA-S; normal cortisol response	Prednisone 70 mg/day (initiated)	Clinical and radiological improvement
November 2023	Polyuria (DI excluded)	Partial regression of CNS lesions	Low LH/FSH; low FT3/FT4 with low TSH; normalised prolactin rhythm	Prednisone 70 mg/day (continued); levothyroxine 25 µg/day; HRT	Stabilisation
April 2024	Weakness, dizziness, impaired concentration	Progression of CNS lesions	Low LH/FSH; low FT4; inadequate GH response (glucagon test)	Prednisone 7.5 mg/day	Disease progression
May–June 2024	Ongoing symptoms	Stable/slightly progressive CNS findings	Multiaxial anterior pituitary hormone deficiencies	Methotrexate 10→15 mg/week; prednisone 10→15 mg/day	Partial response
June 2025	Stable condition	No new CNS lesions	Persistent anterior pituitary hormone deficiencies	Methotrexate 25 mg/week; hydrocortisone 5 mg twice daily; levothyroxine 88 µg/day; rGH 0.4 mg/day	Stable

Abbreviations: ACTH, adrenocorticotropic hormone; CNS, central nervous system; CSF, cerebrospinal fluid; DI, diabetes insipidus; DHEA-S, dehydroepiandrosterone sulfate; FT3, free triiodothyronine; FT4, free thyroxine; GH, growth hormone; HRT, hormone replacement therapy; LH, luteinizing hormone; FSH, follicle-stimulating hormone; MRI, magnetic resonance imaging; NS, neurosarcoidosis; PRL, prolactin; TSH, thyroid-stimulating hormone.

## Data Availability

The original data presented in the study are included in the article, further inquiries can be directed to the corresponding author.
